# Drug-Induced Psychosis: A Drastic Turn After a Kidney Transplant

**DOI:** 10.7759/cureus.100959

**Published:** 2026-01-06

**Authors:** Dalal A Obaid, Maryam AlJasmi, Khawla Y Alawadhi, Sunita Sahu

**Affiliations:** 1 Medicine, Mohammed Bin Rashid University of Medicine and Health Sciences, Dubai, ARE; 2 Pediatrics, Mohammed Bin Rashid University of Medicine and Health Sciences, Dubai, ARE; 3 Internal Medicine, Mohammed Bin Rashid University of Medicine and Health Sciences, Dubai, ARE; 4 Psychiatry, Mediclinic City Hospital, Dubai, ARE

**Keywords:** calcineurin inhibitor, corticosteroid psychosis, drug-induced psychosis, immunosuppressive therapy, kidney transplant, prednisolone neurotoxicity, psychiatric complications, renal transplant, tacrolimus neurotoxicity, transplant psychiatry

## Abstract

Drug-induced psychosis is an uncommon but clinically significant complication in solid-organ transplant recipients, particularly those receiving calcineurin inhibitors such as tacrolimus. Neuropsychiatric toxicity can occur even at therapeutic drug levels, and concurrent corticosteroid use may further increase vulnerability. Early recognition is essential, as symptoms may be mistakenly attributed to metabolic disturbances, primary psychiatric illness, or postoperative delirium, thereby delaying appropriate intervention. We report the case of a previously healthy 34-year-old man with end-stage renal disease who underwent a living-donor kidney transplant and subsequently developed profound behavioral and cognitive changes. Despite no prior psychiatric history, he experienced progressive social withdrawal, auditory hallucinations with running commentary, persecutory delusions toward family members, disrupted sleep-wake patterns, weight loss, and significant functional decline. At the time, he was receiving tacrolimus, mycophenolate mofetil, and low-dose prednisolone for graft maintenance. His family observed a marked deterioration following an episode of acute kidney injury and hyponatremia several months earlier. Mental status examination revealed preserved orientation and attention but prominent psychotic features and poor insight. Tacrolimus trough levels remained within the reported therapeutic range; however, variability in oral absorption and the inability of whole-blood concentrations to reflect the active unbound fraction may have masked neurotoxic exposure. Prednisolone, though prescribed at a low dose, may have contributed synergistically to symptom onset. Alternative etiologies, including primary psychotic disorders, delirium, and mood disorders with psychotic features, were considered but were not supported clinically. This case underscores the importance of maintaining a high index of suspicion for calcineurin inhibitor-related neurotoxicity in transplant recipients who develop new-onset psychosis, even when tacrolimus levels appear therapeutic. In this patient, psychotic symptoms were managed through a multidisciplinary approach that prioritized graft preservation while addressing neuropsychiatric safety. Prednisolone was discontinued, and antipsychotic therapy was initiated, while tacrolimus was continued due to its necessity in preventing rejection. This case highlights that meaningful clinical improvement can be achieved without altering essential immunosuppressive therapy when teams collaborate closely and monitor both psychiatric status and graft function. Early recognition and prompt intervention are crucial to reducing morbidity in this vulnerable population.

## Introduction

The sudden onset of psychosis following the administration of a particular drug or substance raises concern for drug-induced psychosis. Drug-induced psychosis refers to a mental health condition marked by the acute onset of psychotic symptoms that are directly related to certain substances or medications. This occurs when specific agents influence and alter normal brain chemistry. Disruption of the brain’s chemical balance can lead to a range of psychotic symptoms that manifest as abnormal behavior [1].

According to the Diagnostic and Statistical Manual of Mental Disorders, Fifth Edition (DSM-5), medication-induced psychotic disorder is characterized by the sudden onset of prominent delusions and hallucinations that occur shortly after exposure to a medication or substance [2]. The implicated agent must be capable of producing such effects. Delusions are defined as fixed, false beliefs that conflict with reality, while hallucinations are sensory perceptions that are not grounded in external stimuli. These symptoms must not be better explained by another psychiatric or medical condition and should cause significant distress or impairment in social or occupational functioning. Additionally, the symptoms must not occur exclusively during a course of delirium. It is important to specify whether the symptoms developed during medication administration or during withdrawal [2].

The prevalence of drug-induced psychosis varies depending on the medication involved. Among transplant recipients, one of the most common medications linked to this condition is the calcineurin inhibitor tacrolimus. Psychosis has been reported as a side effect in up to 5.2% of patients using tacrolimus [3,4]. Tacrolimus is an immunosuppressive medication primarily administered after solid-organ transplantation to reduce the risk of rejection. It is a macrolide that binds to FK binding protein 12, inhibiting calcineurin and T-lymphocyte signaling. Tacrolimus is one hundred times more potent than cyclosporine and is metabolized by cytochrome P450 3A enzymes. Its levels can vary based on genetic factors, liver function, hematocrit, albumin, and corticosteroid use [4]. Although rare, psychiatric side effects associated with tacrolimus neurotoxicity have been documented in case studies [3,4].

The mechanism underlying these psychiatric symptoms is not fully understood, but calcineurin is believed to affect dopaminergic, glutamatergic, and gamma-aminobutyric acid systems [5]. Disruption of these neurotransmitter pathways is associated with the pathophysiology of common psychiatric disorders such as schizophrenia and bipolar disorder [6].

Another class of medications with rare neurotoxic effects is corticosteroids. These medications are frequently used in transplant patients and have been shown to induce psychosis in some cases. The incidence of corticosteroid-induced psychosis ranges from 1.8% to 62%, with a median onset of 11.5 days after initiating therapy [7]. The mechanisms are not fully understood but are thought to involve enhanced dopamine activity triggered by glucocorticoids [8]. In summary, drug-induced psychosis involves complex interactions with multiple neurotransmitter systems that can become dysregulated by certain medications.

Psychosis related to immunosuppressive medications such as tacrolimus and corticosteroids, including prednisolone, is rare but clinically significant [1]. These agents are essential for preventing organ rejection in transplant recipients. A very rare adverse effect is the development of changes in mood, behavior, and cognition [9]. Differential diagnoses for drug-induced psychosis include primary psychotic disorders such as schizophrenia, in which symptoms generally emerge earlier in life and persist independently of external factors [2]. This case is particularly relevant as it highlights the diagnostic challenge of acute psychosis occurring despite therapeutic tacrolimus levels, with important implications for clinical vigilance in transplant care.

Delirium is another important consideration and is described as a fluctuating disturbance in attention, awareness, and cognition [10]. It can occur secondary to metabolic disturbances, including elevated creatinine levels or uremia [10]. Unlike drug-induced psychosis, delirium typically presents with fluctuating consciousness and disorientation [10]. Patients undergoing major surgery, such as transplantation, may also experience calcium disturbances as rare but serious complications [11]. This makes distinguishing delirium from drug-induced psychosis essential [12]. Depression with psychotic features may also present with hallucinations or delusions, often mood congruent, which helps differentiate it from drug-induced psychosis [2].

Management of drug-induced psychosis requires a multidisciplinary approach. The primary goal is to identify and adjust or discontinue the causative medication while balancing the need for immunosuppression with stabilization of mental health. Antipsychotic medications such as risperidone or olanzapine are commonly used to alleviate symptoms. In some cases, the causative medication cannot be discontinued because of its essential role in preventing organ rejection. Clear communication regarding the importance of maintaining or adjusting the medication regimen is crucial in these situations. Additionally, some medications may have nephrotoxic effects, making careful monitoring and management of kidney function a key aspect of treatment.

## Case presentation

A 34-year-old man with chronic kidney disease (CKD), status post living-donor renal transplant, was brought by his mother and brother for new behavioral changes first noted after the transplant. Since then, he had become quieter and more withdrawn, though initially still able to function at baseline. He had no prior psychiatric history. Post transplant, he was started on standard immunosuppression and remains on tacrolimus (Prograf) 2 mg twice daily, mycophenolate mofetil (CellCept) 250 mg twice daily, prednisolone 5 mg once daily, and adjunctive medications including atorvastatin 10 mg once daily, febuxostat 40 mg once daily, and esomeprazole (Nexium) 40 mg once daily.

The patient had a history of end-stage renal disease and received hemodialysis via a catheter for four months before undergoing a living-donor kidney transplant in April 2019. He was hospitalized in late September 2024 for acute kidney injury and hyponatremia due to dehydration, after which he was diagnosed with stage 2/3a CKD, hyperlipidemia, and hyperuricemia. According to the patient’s mother and brother, this hospitalization was “the turning point, and everything bad happened after it.”

Following this episode, he lost motivation to socialize, stopped meeting friends and speaking with family members, quit his job, and stopped exercising. His brother reported that the patient frequently complained of hearing voices, including his mother’s voice, even when alone in a room. He was often observed muttering to himself and laughing at home. He also made statements such as “the world does not exist” and claimed that he had “many wives and children,” despite being divorced and having no children. He believed his parents were his enemies and expressed a desire to separate his mother and father. His mother reported that he would not listen to them and that she believed he sometimes engaged in behaviors intended to provoke them, such as asking them to find him a wife and then abruptly changing his mind once arrangements were finalized.

The patient had packed all his personal belongings into boxes and had been sleeping on the floor for the past year. He was not sleeping at night and spent most of the daytime asleep. He did not dress appropriately, and his mother had to ensure that he was groomed and wearing suitable clothing before going to public places, as he was often unkempt and would not brush his hair, trim his beard, or pray independently.

He spent most of his time playing video games, and when family members commented on his behavior, he became aggressive and angry. His mother reported that he rarely ate, and when he did, he preferred unhealthy food. Despite her attempts to encourage him to eat, he experienced significant weight loss. As a result of this weight loss, the patient began stating that his chest was too big and that he did not want his muscles anymore, leading him to further reduce his weight against the advice of his nephrologist. This behavior contributed to his hospitalization in September for markedly elevated creatinine levels. Prior to that admission, family members described him as very aggressive and abnormal. They recalled being told by a physician that such behavior could be expected when creatinine levels were very high. Previously, the patient had been an athlete and was described by his family as loving and kind, with his brother noting that he had been his inspiration growing up.

Most of the history of the present illness was obtained from the patient’s mother and brother. During the consultation, the patient denied currently hearing voices but acknowledged that he sometimes hears voices commenting on his actions and speaking about him in a negative way, stating that he tries not to listen to them. He also expressed ongoing beliefs that his parents wanted to harm him and that they were responsible for his divorce.

Mental state examination

Mental status examination revealed a clear conscious state with appropriate grooming but an affect incongruent with the content of discussion, limited speech, linear and goal-directed thought processes, and persecutory delusions toward his parents, accompanied by auditory hallucinations with running commentary. Orientation and attention were intact, though insight into his illness remained poor.

Clinical workup

A focused medical evaluation was conducted to exclude organic, metabolic, infectious, or structural causes that might explain his psychiatric symptoms. No reversible medical conditions were identified. Given the temporal association between initiation of immunosuppressive therapy and the onset of psychotic symptoms, along with the absence of prior psychiatric history or an alternative medical explanation, the findings were most consistent with a diagnosis of drug-induced psychosis related to his immunosuppressive regimen.

## Discussion

Tacrolimus is a calcineurin inhibitor used in solid-organ transplant recipients to prevent rejection through immunosuppression. It has a therapeutic window of 5-20 ng/mL for most adult organ transplant recipients, although target levels may vary depending on the type of organ transplanted, time since transplantation, and institutional protocols [3]. This therapeutic range is intended to maximize efficacy while minimizing neurotoxicity and nephrotoxicity. However, multiple cases of neurotoxicity have been reported even when tacrolimus levels remain within the therapeutic range. Manifestations of tacrolimus-related neurotoxicity include tremor, encephalopathy, psychosis, and rapidly progressive dementia [4].

The pharmacokinetics of tacrolimus are highly complex and variable, which can contribute to toxicity. Tacrolimus demonstrates significant variability in bioavailability, with absorption varying from dose to dose within the same patient due to differences in gastrointestinal absorption. As a result, intravenous administration yields more stable and predictable drug levels than oral dosing. Whole-blood tacrolimus levels are routinely used to monitor therapy and reflect both bound and unbound drug concentrations. However, it is the free (unbound) fraction that mediates both immunosuppressive and neurotoxic effects, and this may be masked by normal whole-blood levels [7]. Tacrolimus trough levels, measured immediately before the next dose, are commonly used in clinical practice as a surrogate marker of drug exposure. In this patient, tacrolimus was administered orally, which may have contributed to variability in absorption. Although trough levels during maintenance therapy were within the reported therapeutic range, these values represent whole-blood concentrations and may not accurately reflect the active unbound fraction, potentially leading to underestimation of neurotoxic risk.

Prednisolone is a synthetic glucocorticoid widely used for its anti-inflammatory and immunosuppressive properties. Corticosteroid-induced psychosis has been well documented, with an estimated incidence of approximately 5-6% of patients [10]. The risk of psychosis is dose dependent and increases significantly when daily doses exceed 40 mg of prednisolone [12]. Milder psychiatric disturbances, such as mood changes and insomnia, may occur in up to 28% of patients [8]. Psychotic symptoms typically emerge within the first few days to weeks after initiation of corticosteroid therapy. Identified risk factors include higher cumulative doses, prolonged treatment duration, prior psychiatric history, advanced age, and female sex [8]. In most cases, symptoms improve with dose reduction or discontinuation of prednisolone, although antipsychotic therapy may be required in persistent or severe cases [13]. Corticosteroid-induced neuropsychiatric manifestations span a broad clinical spectrum, ranging from mood disturbances and cognitive changes to severe psychosis, as described in previous reviews [9].

In this patient, prednisolone was prescribed as part of the immunosuppressive regimen to prevent renal allograft rejection. He presented with auditory hallucinations, delusions, and muttering to himself. Management included discontinuation of prednisolone and initiation of antipsychotic therapy. Quetiapine was started at a dose of 12.5 mg once daily, selected for its efficacy in managing psychotic symptoms and its favorable safety profile in posttransplant patients, with plans for gradual dose titration based on clinical response.

## Conclusions

This case illustrates a medication-induced psychotic disorder in a renal transplant recipient receiving tacrolimus and low-dose prednisolone, emerging after transplantation in a patient without a prior psychiatric history. Calcineurin inhibitor-related neurotoxicity can occur even at apparently therapeutic dosages, and corticosteroids may synergistically precipitate mood, sleep, and psychotic symptoms. Management required a multidisciplinary balance between preservation of graft function and restoration of mental health. This case underscores the importance of early recognition and structured causality assessment of neuropsychiatric symptoms in transplant recipients and demonstrates that timely modification of the offending regimen can be achieved without compromising graft safety when care is closely coordinated with the transplant team.
